# The impact of climate change on the sustainability of wine production and the structure of its consumption in Czechia

**DOI:** 10.1016/j.heliyon.2023.e17882

**Published:** 2023-07-03

**Authors:** Aleksandre Petriashvili, Jiří Mach, Michal Štěbeták, Marie Prášilová, Roman Svoboda, Miroslava Navrátilová, Markéta Beranová, Kamila Veselá, Václav Hofman, Otakar Němec

**Affiliations:** a Faculty of Economics and Management, Czech University of Life Sciences Prague, Kamýcká 129, Prague 6, Suchdol, 165 00, Czech Republic, Czech Republic; b Faculty of Business Administration, Prague University of Economics and Business, Czech Republic, W. Churchill Sq. 4 130 67, Prague 3, Czech Republic

**Keywords:** Agriculture, Climate changes, Sustainable development, Vine yields, Vineyard area, Viticulture, Wine, Wine consumption, Wine grapes, Wine production

## Abstract

Vine-growing for the production of wine constitutes one of the major areas of agriculture of Czechia, and in recent years it has been qualitatively improved. The purpose of this study is to express the effects of climate change on the structure of wine production and consumption in the Czech Republic in connection with the growing local popularity of white wine consumption. The current consumer preferences of wine consumers in the Czech Republic (characterized by the growing popularity of white wines) are not in line with the effects of future climate change associated with the assumption of growing vine varieties suitable for the production of red wines. The methodology of the following study is based especially on the evaluation of statistical data about vine growing and wine production of Czechia and a research investigation about consumers’ preferences in the consumption of wines in Czechia. The effect of long-term climate change in the region are likely to lead to an increase in growing areas, especially in favour of vine varieties suitable for the production of red or rosé wines. The harvest of wine grapes, the hectare yield of grapes and the production of wine in Czechia do not show a significant development trend in the long-term time series of 2000–2019. Thus, in the future, the development of viticulture in Czechia will be influenced mainly by the location of the planted area of vineyards or the development of consumer habits and preferences.

## 1 Introduction

*Grapevine* (Vitis vinifera L.) is one of the oldest crops in the world [1] and its cultivation is reflected in the regional identity of people from different geographical regions [2]. Grapes are grown primarily in areas with favourable climatic conditions, the most important of which are the temperatures during the growing season and the level of insolation [3,4]. These parameters have a critical impact on the content of active substances in the fruit, which determines their taste and suitability for the winemaking process [5]. Vineyards have formed the structure of cultural landscapes of climatically suitable regions for centuries [1].

Vine-growing for the production of wine constitutes one of the major areas of agriculture of Czechia, and in recent years it has been qualitatively improved [6]. As stated by Prokeš and Tomšík [7] “*viticulture and wine industry* in Czechia has undergone over the last twenty years extensive reform and has experienced a significant shift from the quantitative orientation of production towards high quality”. Of the total wine production in Czechia, two thirds are white wine and one third is red wine. These ratios correspond to the ratio of vineyards planted with white and blue varieties. The total area of vineyards in Czechia reached 18,732 ha [8]. The share of the Moravian wine-growing region in the total area of vineyards in Czechia is 96.4%, whereas the share of the Bohemian wine-growing region is a mere 3.6%. For this reason, most of wines produced in Czechia come from the Moravia region [9]. However, Czechia is a country in which the production of wine meets approximately one third of the total demand [10].

Muscio et al. [11] state that the “wine industry is a particularly relevant sector both in terms of its economic performance and its *use of natural resources*.” The growth and production of grapevines grown in vineyards is significantly affected by a number of environmental factors [12]. “Each grapevine cultivar needs a certain amount of cumulated heat over its growing season for its grapes to ripen properly” [13]. Particular cultivars are valuable to farmers in particular applications for their agronomic traits and fruit-quality traits, which together determine the value of the crop and the cost of producing it [14]. Individual cultivars respond to environmental factors differently in terms of the resulting wine quality [15], which is reflected in the concrete and regional-specific characteristics of wines [16].

The specific effects of environmental factors on the quality of wine are called “*terroir*” [17] and known in many wine regions, e.g., France [18]. Van Leeuwen et al. [19] state that terroir is a cultivated ecosystem in which grapevines interact with soil and climate. The main climatic parameters include temperature, rainfall and reference evapotranspiration [19]. „The evaporation process is responsible for transforming a liquid into a gas form“ [20] Vine phenology and grape ripening is mainly driven by air temperature and soil temperature. The soil provides water and minerals to the vine, particularly nitrogen [19]. Van Leeuwen et al. [19] further add that moderate to low vine nitrogen status favours the production of grapes with high quality potential for red winemaking (small berries and high grape skin phenolics). For high-quality white wine production, vine nitrogen status should be at least moderate, because low nitrogen status can impair the production of aroma compounds, in particular those from the volatile thiol family [19]. Research by Cardebat and Livat [21] confirms that “ratings vary among experts, such that some statistically significantly favour wines produced in specific areas, indicating their taste preferences. Thus, preferences matter in expert ratings and would seem to suggest a continental variance.”

„It is now widely accepted that climate change is having a profound impact on the weather systems around the world“ [22]. Ollat et al. [23] state that „*climate change* will have a profound effect on vine growing worldwide“. „The basic idea is that, if the relation between weather and grape quality is known for each grape type in existing growing areas, then it is possible to predict the quality of grapes that would be produced in other locations, or in the same location with a changed climate“ [24]. It can be concluded „that climate change is likely to produce winners and losers, with the winners being those closer to the North and South Poles“ [25].

There are *projections* for warmer, drier, longer-term changes in climate that have the potential to impact all aspects of wine production [26,27]. To reduce negative impacts, vineyard should be relocated, new variety should be developed, alternative sales should be generated (e.g. from vine tourism), and all these changes should be long-time planned and sustainable [28].

These are, in some Bohemian and Moravian regions: rainfall significantly reduced and extreme weather events increased [29]. The wine industry is of particular interest given that evidence suggests temperatures in wine-producing regions around the world have increased since the 1950s [30]. However, temperature increases have actually benefited the industry in terms of better quality and higher prices [31].

Historical records from 1956 to 2021 on bud formation, flowering and ripening of these grape varieties and combined them with global planting data and temperature records from 1880 to 2021 to *calculate models for different global warming scenarios*. The result: with a temperature increase of two degrees, the regions suitable for viticulture would shrink by 56% worldwide; with an increase of four degrees, it would no longer be possible to produce good wines on 85% of the areas [32]. However, the researchers recommend that these figures would be much less dramatic if wine-growers were to cultivate other varieties that are better suited to the new climatic conditions. Such an adaptation could, for example, ensure that “only” 24% of the cultivation areas would be lost with a warming of two degrees. For South Moravia, for example, the scientists suggest growing heat-loving Mourvèdre or Grenache instead of Pinot Noir; in Berounka Region, Mourvèdre could replace Cabernet Sauvignon and Merlot. Colder growing regions in global world (such as New Zealand, the Pacific Northwest of the USA) would survive the two-degree scenario relatively unscathed [33].

*Sustainable agriculture* involves obtaining healthy and quality foods, conserving natural resources and preserving biodiversity [34]. Escoto et al. [35] state that “sustainability plays an important role in society by improving long-term quality of life, including future generations, seeking harmony between economic growth, social development and the protection of the environment”.

According to its principles, the economic aspect of the development should perceive society and the natural environment not as its inhibitors but rather as stimulants [36,37]. Prus [37] further adds that sustainable development in agriculture means such programming of farming production so that it makes reasonable use of natural resources and the environment. It provides sufficient amounts of food while maintaining its high quality.

*The research gap* is the opportunity to examine the relationship between the current growing popularity of white wine consumption in the Czech Republic and future climate change in the given area, which will lead to the need to grow vine varieties suitable for the production of red wines.

*The purpose of this study* is to express the effects of climate change on the structure of wine production and consumption in the Czech Republic in connection with the growing local popularity of white wine consumption. The article should also contribute to emphasizing the need for rapid implementation of changes in the structure of wine production that would correspond to the speed with which climate changes occur.

In order to evaluate the fulfilment of the research goal, the following research *hypothesis* was established: “The current consumer preferences of wine consumers in the Czech Republic (characterized by the growing popularity of white wines) are not in line with the effects of future climate change associated with the assumption of growing vine varieties suitable for the production of red wines.”

## 2 Methods

The methodology of the following study is based especially on the evaluation of statistical data about vine growing and wine production of Czechia and a research investigation about consumers’ preferences in the consumption of wines in Czechia. The source of data for the creation of the time series of selected indicators of viticulture and grape vine is the CZSO [8] and the Ministry of Agriculture of the Czech Republic [38].

### 2.1 Methodology of time series evaluation

The methodology of scientific research contained in this article was based on a description of the development of the monitored quantities, as well as the subsequent comparative analysis of the time series of selected indicators and their evaluation. These were specific indicators of the economic performance of the viticulture and grape vine sector, namely the grape harvest, the area of vineyards, and wine production in Czechia.

The description of the development of the time series of yield-generating indicators of vine growing and wine production in the conditions of the Czech Republic was performed using exponential smoothing methods. The created model was then used to predict the evaluated indicators. The solution procedure was based on the assumption that the next value in the time series is affected by the previous one. However, the new value in terms of time has more weight in the model (formally more important) than the value a period older. The prediction was performed using the second-degree exponential smoothing method (Holt's method), which was extended by a damped trend [39]. The original Holt's model was enriched by the phi coefficient, phi∈ <0; 1>. The phi coefficient can dampen the linear trend to the value to which the time series tends to converge:

(1) $$\alpha_{Holt}=\alpha \left(2-\alpha \right);\gamma_{Holt}=\frac{\alpha }{2-\alpha },where\;\alpha \in <0;1>,\gamma \in <0;1>,phi\in <0;1>$$

The Mean Absolute Percentage Error (M.A.P.E.) was decisive for the final choice of model coefficients (alpha, gamma, phi). The choice of model was based on the usual rule, which considers a case with a value of M.A.P.E. below 5% to be a very good prediction model and a case with a value of M.A.P.E. below 10% to be a satisfactory model [40]:

(2) $$MAPE=\frac{100}{n}\times \sum_{i=1}^{n}\frac{\left|y_{i}-u_{i}\right|}{y_{t}}\text{,}$$

where n … ….the number of members of the time series,

y_i_ … ….empirical values of the time series,

u_i_ … ….theoretical values of the time series.

### 2.2 Methodology of evaluation of questionnaire survey

To find out the interest of consumers in wine consumption in the Czech Republic, a questionnaire survey was conducted on a sample of 570 respondents. The underlying data were processed by statistical methods from the field of qualitative features. The χ2 independence test was used for the dependence of the relationships between the selected questions in the questionnaire. The test criterion is based on Pearson's chi-square statistics [41]:

(3) $${Χ}^{2}=\sum_{i=1}^{r}\sum_{j=1}^{s}\frac{{\left(n_{ij}-n_{ij}^{\prime }\right)}^{2}}{n_{ij}^{\prime }}$$

where *n*_*ij*_ the empirical frequency,

*n′*_*ij*_ the theoretical frequency,

*i* = *1, …, r*, where *r* is the number of the *A* variable varieties,

*j* = *1, …, s*, where *s* is the number of the *B* variable varieties.

The *χ*^*2*^ test criterion is governed by *χ*^*2*^ distribution with *[(r-1) (s-1)]* degrees of freedom. To determine the dependence between the characters, the p-value was used, which is directly compared with the selected level of significance α (here α = 0.05). If the p-value is less than α, then the dependence on the level of significance α has been demonstrated.

The strength of the relationship between the A and B variables has been established using the Cramér contingency coefficient *V*:

(4) $$V=\sqrt{\frac{\chi^{2}}{n\left(q-1\right)}}$$

where *n* is the size of the sample, *q* = *min (r, s)*, *V∈* < *0;1>*.

### 2.3 Climate models

This article seeks to verify the fact that despite the preference for white wine among the Czech population (as our research has shown in Chapter 3.5), potential climate changes highlight the need for a significant shift of wine-growing areas to the northern parts of the country and, subsequently, towards the cultivation of vine varieties suitable primarily for red wine production.

Climate models focused on the future of vine growing are based primarily on the Huglin index, which uses the daily average and maximum temperature of the area during the growing season (from early April to late September). At present, there are around 40 climate models from various world institutions. Five were selected, the best known being -CNRM (National Center for Meteorological Research - France), which is a more optimistic model, and HadGEM (Hadley Center Global Environment Model - England), which is a more pessimistic model. Two warming variants were processed for each period. A pessimistic scenario for the evolution of greenhouse gas concentrations in the atmosphere RCP 8.5 (watts per m^2^) and a more optimistic scenario RCP 4.5 (watts per m^2^), which assumes that the CO_2_ concentrations could by stabilized at a lower level during the 21st century.

## 3 Results

### 3.1 The impact of climate change on the structure of cultivated vine varieties in Czechia

**Fig. 1:**
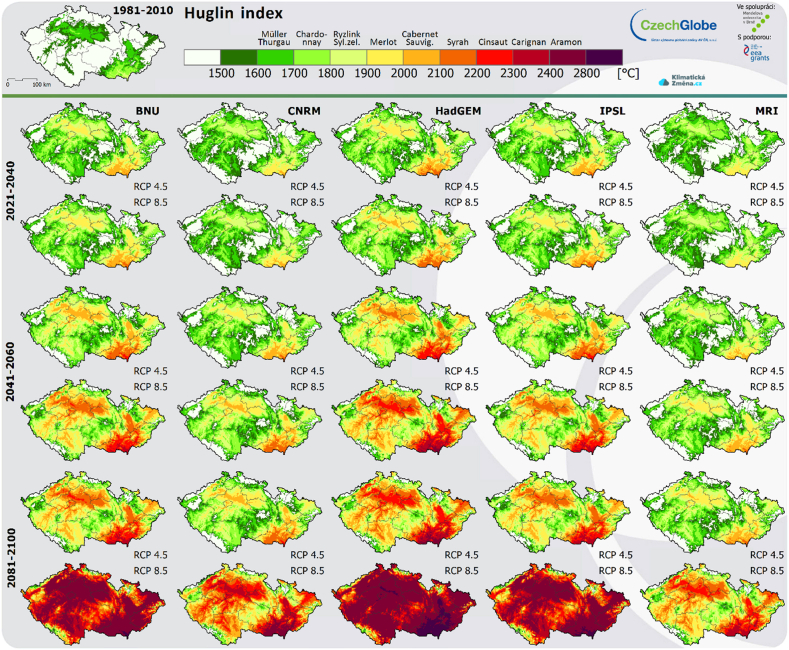
Huglin index for Czechia [42].

In Fig. 1, on the top left can be seen Czechia in the climatic conditions that have been used to so far. To the right of the map of the Czech Republic, a colour scale with the names of individual vine varieties is shown, expressing the suitability of their cultivation at a certain value of the Huglin Index. The rows below then show the predictions calculated using the major climatological models (BNU, CNRM, HadGEM, IPSL and MRI) for the entire 21st century. Each period and each model then has two variants.

Therefore, for example, in the years 2041–2060, according to the optimistic variant (RCP 4.5) of the CNRM model, it will be possible to grow the popular Ryzlink rýnský variety in Prague vineyards; according to the HadGEM model, this will no longer be possible and it will be appropriate to grow, for example, Cabernet Sauvignon. For the pessimistic variant of both mentioned models (RCP 8.5), the vineyards in the territory of the capital city of Prague would be suitable only for the cultivation of red varieties, e.g. Merlot or Cabernet Sauvignon.

Although the average temperature is not expected to rise by more than 0.8° Celsius over the next 20 years, the change is more dramatic in the sum of average and maximum temperatures over the whole year. Just as important as the full-year total will be the year-to-year variation in their values and how large the individual extreme fluctuations will be. Overall, however, it is clear that it will continue to warm up and therefore the value of the Huglin index will increase (see Fig. 1), which is why southern Moravia will move from areas primarily suited to white varieties to red varieties [33]. However, it depends on the specific location of the vineyard, how much sun there is, what kind of exposure it has, and also on the preference of customers as to the character of the wines they want to get from that location.

The record-breaking early harvest of vines in 2018 brought with it both enthusiastic responses and many questions for the future. Of course, global warming is also affecting Czechia. Over the last 60 years, the average temperature in Moravia has increased by approximately one and a half degrees Celsius. Experts agree that warming will continue, and so wine-makers and wine lovers rejoice - Czechia will become a lucrative wine region. Climate models using the Huglin index (see Fig. 1) suggest this.

Wine-makers are generally satisfied, especially with the quality of red wines. Twenty years ago, if the Frankovka wine from the area of Modré Hory of Moravia reached 20° of sugar content, it was an exception; currently 22° is a common value. At the same time, Moravian wines from varieties that until recently were only known to Czech consumers from the southern regions [43] are appearing on the market more and more often.

The vineyards have been bearing grapes for about thirty years, so wine-makers will have to consider such a period in advance when planting vines. If thermophilic vine varieties are now appearing in Moravia, it is proof that wine-makers believe or have believed in climate change some time ago. However, it should be borne in mind that the geographical location of the country will not change, so there may continue to be extremely low temperatures during the winter, even in places that can be devastating for thermophilic southern vine varieties located there. This should be taken into account when selecting non-traditional vine varieties.

Due to the acid in the grapes, some earlier varieties are beginning to be harvested with late ones at the same time. For example, wine-makers from Château Valtice had to invest in technological equipment in the winery and speed up the harvesting of grapes. Today, according to them, it is no longer a question of obtaining higher sugar content, but of trying to maintain a balance between sugar and acid [44].

Drought is often spoken of as a major coming problem, especially for farmers. Although, unlike other crops, vines are slightly more resistant to drought, this clearly applies to them as well. For example, when a certain temperature threshold is exceeded, the grape stops to photosynthesize to retain enough water. If the temperature rises even more, the grapes will change their taste, for the worse. „Grape growing regions are facing constant warming of the growing season temperature as well as limitations on ground water pumping used for irrigating to overcome water deficits“ [45].

### 3.2 Economic performance of viticulture in Czechia

**Fig. 2:**
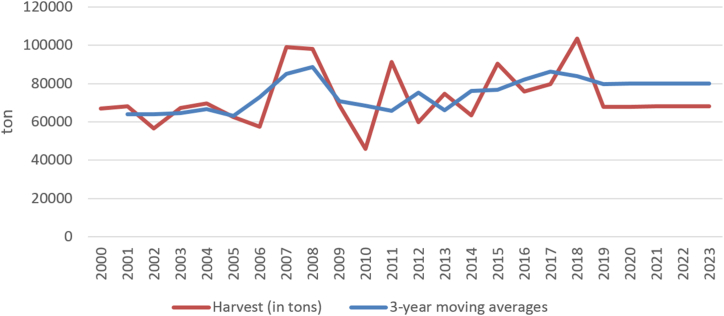
Harvesting grapes in Czechia (in thous. tons) [38].

**Fig. 3:**
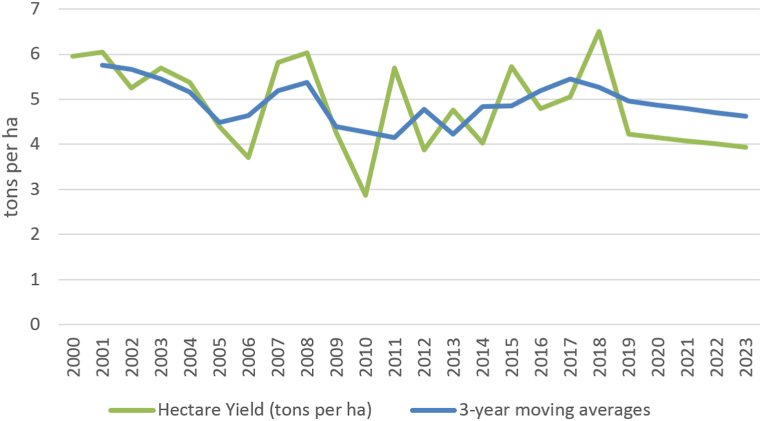
The yield of vines in Czechia (in t/ha) [38].

**Fig. 4:**
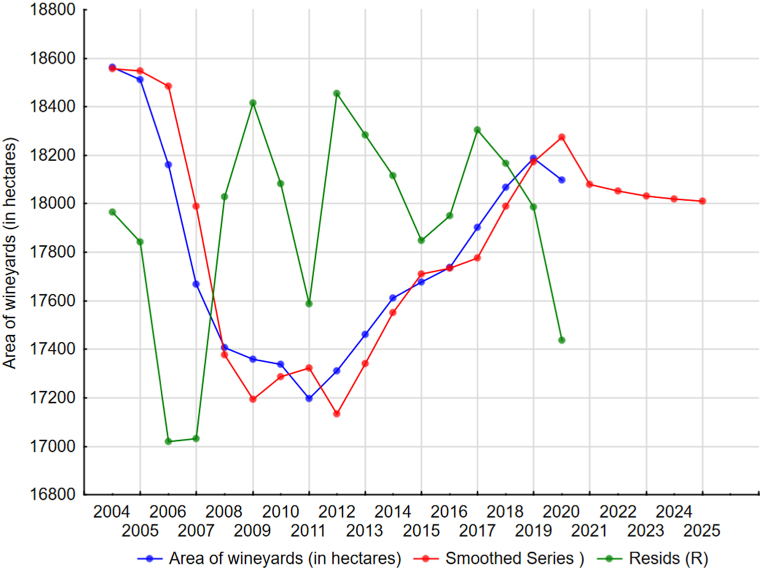
Predicted vine acreage (in hectares) [8].

The harvest of grapes, the hectare yield and the production of wine do not show a significant development trend in the long-term time series of 2000–2019. Therefore, the trend for all indicators is described using moving averages. Short-term forecasts are shown in Fig. 2, Fig. 3, Fig. 4 and document the future sustainable development of the economic performance of viticulture and grape vine growing in Czechia.

#### 3.2.1 Harvesting grapes and the yield of vines in Czechia

Due to August and September rains and the threat of rottenness in 2014, wine-growers harvested a big proportion of crops, especially with early variety, earlier. With an average yield of 4.3 tons per hectare the wine growers harvested 63,500 tons of grapes (see Fig. 2, Fig. 3).

In October 2014, the European Commission granted winemakers from Czechia and other countries an exception under which due to rainy weather they may increase the sugar added in wines more than is required by EU rules. The yields of vines in 2015/2016 were above average at the level of 5.7 tons per hectare and the grapes were in very good condition (see Fig. 3). According to CZSO [8], 90,600 tons of grapes were harvested in Czechia in 2015, which is the most since 2011.

The wine-makers agree that, in terms of the amount of grapes harvested, the harvest in 2016 was marked by spring frosts, which irreversibly destroyed most of the grapes in some areas of Moravia. On average, Moravian vineyards suffered a 30% crop loss. However, the rest of the vegetative period of the vine was good, so wine-makers enjoyed the good quality of the harvested grapes, for all varieties.

According to a report issued by the Wine Growers Association of the Czech Republic (2021), approximately 605 thousand hectolitres of wine were obtained from grapes harvested in Czechia in 2017. The yield of grapes was around 5.2 tons per hectare (which is four percent more than in 2016). The ten-year average yield is 5.1 tons (the ten-year average yield over the last decades has fallen from 6 to 5.1 tons per hectare). At the same time, for example, in Germany the long-term average yield of grapes was doubled and in Austria it was higher by about 40%. The average sugar content was around 20.9 °NM (standard must meter - indicates the sugar content per kg unit per 100 L of must (Act No. 321/2004 Coll. on Viticulture and Wine-growing in the Czech Republic 2021 [46]), which was 0.5° more than the ten-year average. In terms of diseases and pests, nothing significant happened in the summer of 2017, the only problem was drought and lack of precipitation. Although the damage was slightly larger than in 2016, the vines regenerated quite a lot, so in the end the yield of 2017 was slightly higher than the previous year.

According to South Moravian wine-makers, the wines from the 2018 harvest, and especially red wines, are an exceptionally good vintage. The unusual course of the season (a very early harvest) gave the grapes different characteristics than usual. Just like they have in France or Spain. The hot and dry summer filled the grapes with higher sugar content and, conversely, lower acids than are typical for Moravian grapes.

The harvest of grapes in Czechia in 2019 was up to 20% lower than in the previous year, but the quality of that year's wine was above average. Thanks to the extremely hot June and the changing temperatures in August, the grapes had ideal conditions for growth and gradual ripening. The rains at the turn of August and September did not cause any major damage either; the grapes were therefore in very good condition. Also, disease and extreme weather fluctuations avoided the vineyards.

The yield of grapes in 2021 is estimated at a similar level as in 2020, which is good and wine-makers are satisfied with it.

The long-term development (2000–2019) of the indicator of grape production and their yields per 1 ha shows an essentially unchanging trend. Year-on-year changes in indicators are caused by weather fluctuations in individual years (drought or rain, etc.).

#### 3.2.2 Area of vineyards in Czechia

In 1996, there were approximately 12,650 ha of vineyards in the Czech Republic, of which 12,000 ha were in Moravia and 650 ha in Bohemia. From 1990 to 1993, the annual decrease was 600–700 ha of vineyards, in 1994 it was just over 450 ha.

After Czechia joined the EU, quotas were issued, which determined the maximum area of vineyards. New vineyards were planted hastily so that the area was as large as possible before the stop state. Czechia thus gained an area of just over 18.5 thousand hectares (see Table 1). In addition, 2% of the so-called state reserve was added. However, it was almost not used, and on the contrary, the number of active vineyards decreased. As Tibor Nyitray, the president of the Czech Winegrowers Association, points out, “there were several reasons, but perhaps the most important was the very difficult access to subsidies or money. It is necessary to realize that the planting of 1 ha of vineyards costs a million crowns” [9].

**Table 1:** Development of vineyard areas in Czechia since accession to the European Union (in hectares) [9].

Year	Vineyard areas in ha
2004	18,564
2011	17,198
2018	18,068

From the beginning of 2016, EU countries can expand their vineyards annually by one percent of the planted area. In Czechia this is approximately 170–180 ha. In the first years, winegrowers were interested in new planting of vines, but the overall offer was not exhausted. For example, in 2017, growers could apply for up to 177 ha. There were 189 applicants who wanted a permit for an area of 152 ha. A year later, they applied for 226 ha, exceeding the quota by 48 ha. In 2019, there was even higher interest. In the previous year, 2018, it was enough to reduce the requirements that did not fall into the vineyard lines, therefore in 2019 they were also reduced in the vine-yard lines. In the Czech Republic, vines are now grown on about 18,000 ha. The current interest in vines is mainly because the industry is thriving and wine-makers believe in the future of planting vineyards and wine production, and subsidies are much more accessible than 14 years ago.

The prediction of the harvest area of the vine was performed using the Holt model of exponential smoothing (equation (1)) and responds to European and national subsidies to this sector. The lowest values of MAPE (0.6123%, equation (2)) are shown by the prediction model with the parameters alpha = 0.9, gamma = 0.9 and phi = 0.8 (see Table 2 and Fig. 4).

**Table 2:** Development of vineyard areas in Czechia since accession to the European Union (in hectares).

Year	Vineyard areas in ha
2021	18,078.21
2022	18,051.29
2023	18,032.44
2024	18,019.25
2025	18,010.02

### 3.3 Wine production in Czechia

Despite its tradition in viticulture and wine-making, the Czech Republic is one of the few countries where wine production is not enough to cover its consumption. There is a surplus of wine on the world market, especially cheap table wine. In Europe alone, around 250 million hectolitres of wine are produced each year, with annual consumption reaching around 170 million hl and still declining. In the EU countries, the decline is on average 2 million hl per year. A certain part of this surplus, especially in the past, ended up on the domestic market, where it was often price-advantaged over domestic products thanks to state subsidies.

Approximately the same amount of wine as domestic production is imported to the Czech Republic every year. According to the Wine Growers Association of the Czech Republic [9], the domestic production amounted to 336 thousand hl in 1995. This is less than half of the production in 1994, when approximately 750,000 hl of wine was produced in the Czech Republic.

In Czechia, grapes were harvested at 1.7 billion crowns (65 mil. EUR) in 2011, which is the double of the year 2010. This year winemakers produced about 670,000 hL of wine which represents approximately one third of the annual wine consumption in Czechia [9].

Local production of wine in the season of 2012 fell by more than a quarter, roughly to 470,000 hL of wine. The production of 2012/2013 accounted for 315,000 hL per white wine and 155,000 per red wine. Average annual consumption of wine reached twenty litters per person [9].

**Fig. 5:**
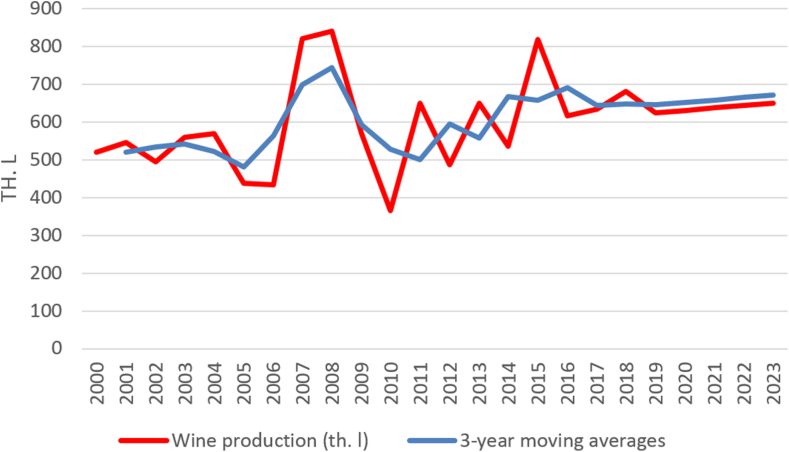
Wine production in Czechia (in thous. hl) [38].

Winemakers in Czechia produced in season 2014/2015,536 thousand hectolitres of wine. It is about 17.5% less than in the previous season. The world-wide production of wine has declined by 3.2%–267 million hectolitres in 2016, and cultivation of grape vines decreased mainly in France and Argentina. The main reason for this was bad weather, as has been reported by the International Organization of Vine and Wine [47]. In Czechia, production in 2016 reached 686,000 hL compared to 819,000 hL in 2015, which was a record-breaking low (see Fig. 5) [47].

The production of wine in 2021 in the Czech Republic will be similar to that in 2020, when it exceeded 600 thousand hectolitres [48]. Compared to the previous five years, this is the second highest volume after 2018. This is the total production, including both domestic production and imports. However, the data of the Wine Fund do not include small wine-makers with a production of less than 1000 L who produce wine for their own consumption and are not obliged to report data on their production.

### 3.4 Total wine consumption in Czechia

The purchasing power of the population of Czechia and the associated growth of the wine market has been constantly increasing in the last twenty years. Despite this apparent increase, wine consumption per capita in Czechia is below the European average, but is expected to reach this level in the short term.

In 1920 the consumption of wine per person was only 5 L and after World War II about 6 L. In the revolutionary year of 1989 it was between 13 and 14 L. The reported consumption of wine in the Czech Republic in 1995 was about 1.2 million hl per year, i.e. 12–15 L per person. In 2008, the Czechs drank an average of 16.3 L of wine per person. 16.9 L was the consumption of wine per person in 2016 in Czechia.

**Fig. 6:**
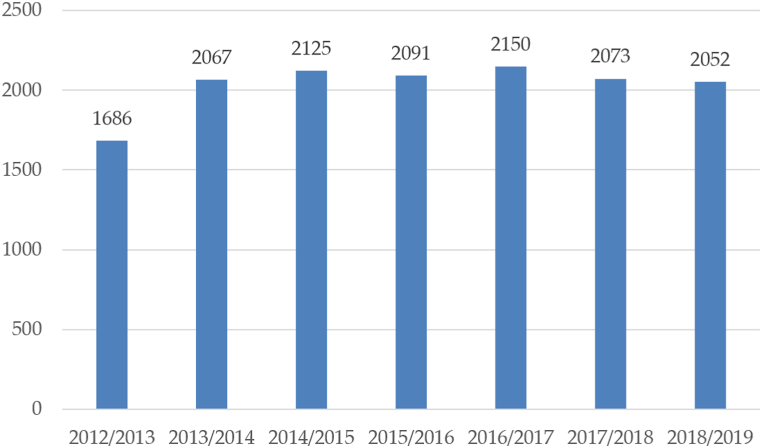
Total consumption of wine in Czechia in thousands of hl [9].

The total consumption of wine in Czechia in the wine year 2018/2019 was 2052 thousand hectolitres (see Fig. 6) [9]. Consumption of wine per capita reached 20.4 L per person per year in 2018, which represents an increase of 25.15% compared to 2008.

At present, around 210 million litres of wine are consumed annually in the Czech Republic. Among them are wines from domestic vineyards, imported wines, and wines that are produced in the Czech Republic, but the grapes are from abroad. A litre of wine costs 60 crowns on average and imported wines are generally cheaper [48]. In the Czech Republic, about 60% of the wine consumed comes from abroad. These are often cheaper surpluses, but the country is dependent on imports.

Local wine production, which is preferred by consumers, has not satisfied even a third of the demand for wines in Czechia in recent years. It is a great challenge and opportunity for wine producers from Moravia and Bohemia. Switzerland could serve as a good example, as the export of local wine is practically non-existent there and all production is consumed on the own market at prices that are comparable and often higher than the prices of imported wines. Syrovátka et al. [49] add that, reasons for the higher consumption of wine in the Czech Republic are seen in a change of the lifestyle, which is connected with the prevailing decline from spirits with higher alcohol contents.

In 2021, wine-makers also rely on customer solidarity. This was also confirmed by a survey among clients of Bohemia Sekt. Over 80% of respondents said that after loosening the restrictions, they will support the Czech companies by purchasing their products [50].

### 3.5 The research on wine consumption in Czechia in 2020

The popularity of wine in Czech society is confronted with the popularity of beer. Beer is a traditional everyday drink, especially for the male population. In recent years, however, beer consumption per capita has been declining and wine consumption has been increasing. The Czech market offers a relatively wide range of wines of both domestic and foreign production.

**Fig. 7:**
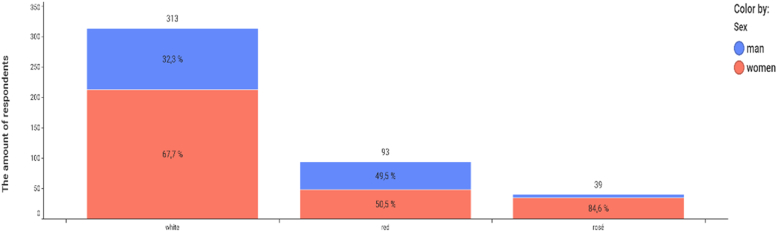
Wine consumed by classification and gender of consumer.

To find out the consumer interest in wine, a questionnaire survey was conducted in the Czech population as part of the research. The respondents were residents aged 18 and over from all regions of Czechia. The questionnaire survey took place in the spring of 2020 in the form of an internet research that took place on the CULS server in Prague, which was publicly accessible. The survey and was attended by 570 respondents (417 women, 153 men), of whom 78.07% consume wine. More women (65.62%) drink wine than men. Fig. 7 shows that the Czech consumer prefers white wine.

**Fig. 8:**
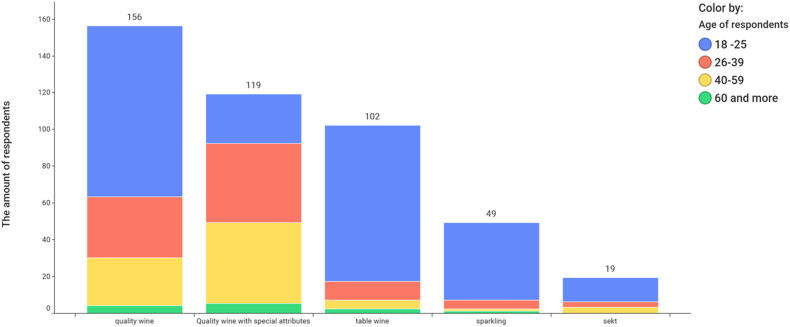
The most frequently consumed type of wine product and the age of the consumer.

While red wine is popular with both men and women, rosé wine is clearly sought after by women. Table 3 shows the results of statistical testing using formulas No. 3 and 4. The research showed that the age of the respondent is a very important factor in the consumption of wine by type of wine product (Fig. 8, Table 3). The age group of 26–39 years has stable incomes to provide for themselves and their families, travels often and can afford to consume quality and special wines. The test criterion χ2 (χ2 = 106.2946), describing the dependence of the type of wine product and the age of the consumer, is almost certainly statistically significant (see Table 3) and this dependence is moderately strong (V = 0.3456). Statistical significance was also demonstrated in the relationship between gender and wine category (χ2 = 16.2331), with women preferring red wines (Table 3, Fig. 10).

**Table 3:** Statistical analysis of relationships.

Relationship	Test criterion χ2 (equation (3))	p-value	Cramér's V (equation (4))
Wine preferences by classification and gender	16.2331	0.0003	0.1910
Type of wine product and age of the consumer	106.2946	0.0000	0.3456
Price of wine and gender	3.5729	0.3114	0.0896
Wine preferences according to sugar content and age	36.5760	0.0000	0.2027

**Fig. 9:**
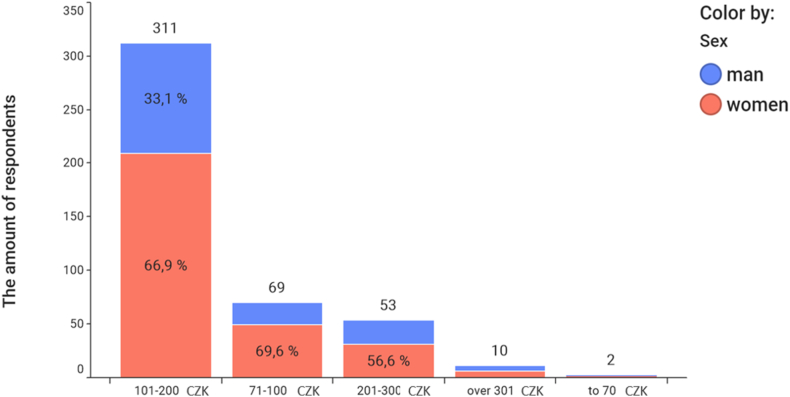
Usual price of purchased wine and gender.

Wine is most often sought after by young people in the age category of 18–25. The price of wine does not play a significant role in purchasing (see Fig. 9).

**Fig. 10:**
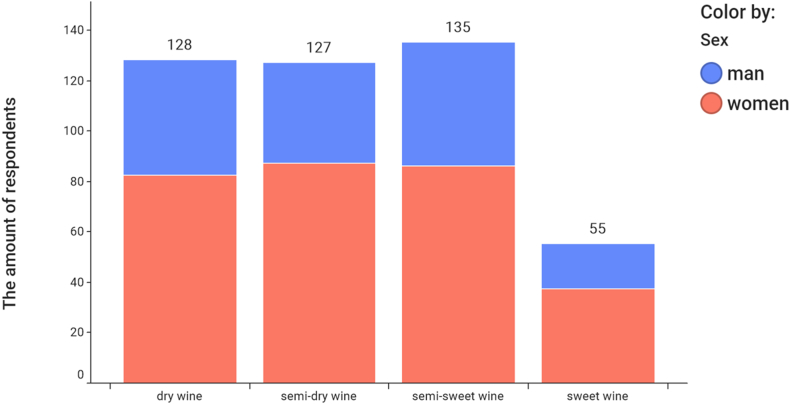
Wine preferences according to sugar content and gender.

The Czech consumer most often buys wines in the price range of 4–8 EUR (101–200 CZK). Men and women react equally to the price of wine (see Fig. 10). The situation is different when evaluating the relationship between wine preference according to sugar content, gender and age category (Table 3).

Czechia is specific in that, compared to the rest of countries, wines with residual sugar are very popular. In recent years, however, this has changed a lot and Czechs are increasingly choosing and consuming drier and also more interesting wines (see Fig. 10).

As demonstrated by the research of Tempere et al. [51], the complexity of wine flavour is strongly linked with grape quality, depending on grape-growing practices and wine-making procedures, winemakers are concerned to anticipate some stabilization of consumer preferences for specific, intrinsic, wine characteristics.

### 3.6 Consumer behaviour research - the Wine Fund of Czechia

The consumer behaviour research, which was carried out by the Wine Fund of Czechia, presents the basic characteristics of wine consumption in Czechia in 2016.

Moravian and Bohemian wines are a clear priority for consumers. 60% of Czechs prefer them, while less than a tenth of consumers prefer foreign production. “Wines from Moravia and Bohemia are closest to us and we prefer them mainly thanks to their better and generally satisfactory taste and high quality. However, we are also happy to purposefully support Moravian and Bohemian producers with our purchases,” Jaroslav Machovec, director of the Wine Fund [48], which has been commissioning regular consumer behaviour research since 2006, commented on the research results.

The research also showed that the Czechs liked the Pálava, Rulandské šedé, Chardonnay, Sauvignon or Tramín červený varieties of white wines the most. Among the red wines, Modrý Portugal leads, followed by Frankovka and Svatovavřinecké. There is demonstrably great interest in Pálava, not only on the local market, but also abroad. The variety is easily recognizable for consumers and interesting for its distinctive aroma. At the same time, as a local product bearing the name of Pálavské vrchy, it often serves as a souvenir [43].

## 4 Discussion

Due to economic interest, the impacts of climate change on wine production have been already studied by many authors (e.g. Refs. [[52], [53], [54], [55], [56], [57]]). The following main topics can be considered essential for the discussion on the future sustainable development of viticulture and wine-making in the Czech Republic:

### 4.1 Climate change and its impact on viticulture and wine-making

More frequent heavy rain events and longer dry phases favour the establishment of new and spread of established pests. Rising temperatures during the ripening phase change the grape constituents. In order to be able to produce typical wines of the highest possible quality in the future, winegrowers will have to adjust various parameters, ranging from variety selection, soil cultivation, irrigation, cultivation work and plant protection to oenological strategies such as acid management.

For example, changing climatic conditions with increasingly long dry phases are causing water shortages and consequently a nitrogen deficiency in the soil during the growing season. This deficiency impairs fermentation and thus aroma development. If heavy rainfall then suddenly makes high amounts of nitrogen available to the vine, this has negative consequences for grape health.

Jagosz et al. [58] state that “climate changes lead to a rise in air temperature, which significantly increases the water needs of plants”. More frequent heavy rain events and longer dry phases favour the establishment of new and spread of established pests. Rising temperatures during the ripening phase change the grape constituents. In order to be able to produce typical wines of the highest possible quality in the future, winegrowers will have to adjust various parameters, ranging from variety selection, soil cultivation, irrigation, cultivation work and plant protection to oenological strategies such as acid management. The climate suitability for wine grapes’ cultivation is at greater risk than that of other crops due to both short-term climate variability and long-term climate changes [59,60]. However, there are strategies for adaptation: by switching to wine varieties that are more resistant to heat and drought, the losses can be at least partially compensated. By growing other varieties, the damage in the two-degree scenario can still be limited to 24%. For four degrees, the damage is limited to 58% for cultivated areas even with adapted grape varieties.

For wine-growing areas in southern Moravia, however, this countermeasure would hardly help, as they already plant the most heat-resistant grape varieties. Vines in cooler growing regions like Czechia, on the other hand, would survive the two-degree warming relatively unscathed and could even benefit by growing late-ripening varieties like Merlot.

Vintners in central Moravia would benefit significantly, as they could plant new wine varieties for the first time that would not have survived in the previous cold climate. The number of cultivable wine varieties would even increase from zero to five. The researchers also point out that practices such as increased irrigation or shade created by tarpaulins can protect plants, but only if global warming is low.

In principle, the emerging risks for Bohemian and Moravian soils as a result of climate change are manageable according to current knowledge. In order to practice quality viticulture at all, certain conditions must be present: light, warm, dry soils with a quantity of organic matter that is not too high, but also not too low, to allow healthy vegetative growth. Grapes are extremely sensitive to climatic changes and here especially to those affecting temperature.

Van Leeuwen et al. [61] recommend that terroir expression at specific sites might be maximized by choosing appropriate plant material in relation to soil and climate, by acting on manageable parameters like vine water and nitrogen status, or by implementing canopy management to modify microclimate in the bunch zone.

### 4.2 Prediction of the area of vines in Czechia

The above statistical prediction of the area of vines in Czechia shows that between 2021 and 2024 it should increase from 16,277.17 ha to 16,453.05 ha (i.e., by 1.08%), which expresses its sustainable development in the coming years. Therefore, there should be no loss of vineyard area during this period if the current conditions for its use in grape production continue to exist.

The change in the expansion of vineyards was welcomed by wine-makers in Czechia. “We are happy to be able to expand the vineyards every year,” says Nyitray (the president of the Czech Winegrowers Association) adding that the opportunity to expand the planted area by one percent is also satisfactory [43].

According to him, there were also proposals for greater expansion. “From our point of view, we are able to plant vines annually, so that we can subsequently include the increased production in the market, up to a maximum of 2.5%,” points out Nyitray [43].

### 4.3 Change in wine consumption

As follows from the statistical data on wine consumption in Czechia (Fig. 6), there has been no significant change in wine consumption in the last monitored years. It ranges from 2052 thousand. hl. in 2018/2019 up to 2150 thous. hl in 2016/2017. The difference between the extreme values of wine consumption was 98 thousand. hl, which in relation to consumption from 2016/2017 represents only a 4.6% change. This relative stability of the development of wine consumption, thus supports the idea of sustainable development of wine production in Czechia with regard to the perspective of stable wine sales.

A similar topic is addressed by Dubois et al. [62] who document how the COVID-19 crisis has affected the drinking behaviour of Latin European wine consumers. The number of people who maintained their wine consumption frequency is significantly higher than those who increased or decreased their consumption. Wine consumption frequency held up better than other types of alcohol (beer and spirits).

According to the Copa Cogeca agricultural organization [63], the harvest of grapes in 2021 in the countries of southern Europe will be significantly lower. The organization expects production of over 117 million hectolitres, which is a year-on-year decrease of 18%. Spring frosts have decimated about 30% of cultivation in France and northern Italy. The losses were also exacerbated by hail, drought and disease. “The average over the last 10 years for Italy, France and Spain is 135 million hectolitres. The decrease against this average is 13.7% [63].

For the domestic consumer, this means that for selected batches and the current vintage, this may affect the price and quantity of the wine. On the other hand, Covid has affected countries in the south as well in the past two years. With their production, the local wine-makers definitely did not have the opportunity to sell everything, so this year's decline can be offset by older vintages. Again, however, it is a question of price, which may be higher than for young wines.

Both surveys have shown the prevailing popularity of white wines among consumers in Czechia, regardless of their age. In terms of the structure of consumers, both surveys have shown that wine is most often sought after by young people aged 18–25, with a significant group of younger women under 34 who drink almost a quarter of all wine in Czechia. Wine is generally drunk more by women than men.

### 4.4 Findings of German wineries on climate change

What connects Czech and German wine-makers is not only the location of the vine-yards on a similar geographical parallel, but especially the significant popularity of white wine consumption by locals, most often Ryzlink varieties. Therefore, it is also beneficial to compare the experience of both national viticultures.

Climate change resultant hazards have become a major threat to farming, food production systems and agricultural sustainability globally [64]. German wine growers recognized the force of climate change as floods swept through the Ahra and Moselle valleys; similarly, Moravian wine growers became aware of this power when, in 2021, a tornado destroyed vineyards in the Mikulov wine-growing sub-region. Other German wine-makers see and feel it differently and are also not enthusiastic about it, because they have to change a number of procedures. For example, the years from 2017 to 2020 were drier, so there was a problem with water. The grapes are also sweeter, making the wine more alcoholic, although the new trend is the opposite: consumer demand for wines with less or no alcohol is rising. Colder regions, such as ours, where it is common to have less sugar and therefore less alcohol, benefit from climate change.

“The biggest problem with climate change is drought,” said Stefan Doktor, a leading Rhine Riesling grower at the Schloss Johannisberg winery in Germany. “An increase of 1 °C means a 25% increase in evaporation.” Therefore, the winery built an irrigation system on the upper part of the vineyard.

“Czech-German relations are currently as good as ever“ [65,66], which allows the Czech Republic to put into practice the aforementioned findings of German wine-makers. However as is stated by Van Leeuwen et al. [67], in each location environmental conditions are different, so there is no general recipe that can be applied everywhere. This explains why plant materials and viticultural techniques vary so much across winegrowing regions of the world.

### 4.5 Verification of the validity of the proposed hypothesis

Based on the analysis of data obtained from the database of the Czech Statistical Office, a study of professional publications, reports of the Czech Winegrowers Association on the state of viticulture and grape vine growing in Czechia and findings on the relationship between wine production and consumption, it can be stated that the established hypothesis was confirmed.

The sustainable development of viticulture and vine growing in Czechia depends not only on the preferences of wine consumers, but may also be significantly affected by the impact of ongoing climate change, which would be reflected in changing the composition of vineyard planting in favour of red vine varieties.

To achieve this goal, it is appropriate to implement the following recommendations.

#### 4.5.1 Resulting recommendations for winegrowers

• Like other farmers in Czechia, winegrowers should take steps to retain water in the soil; among other things, plant trees near the vineyards to maintain water so as not to affect the vegetation of the vine.
• Given that, since 2016, EU countries have been able to expand vineyards annually by up to one percent of the planted area, it is advisable for Czech wine-makers to take full advantage of this opportunity. These are mainly areas of Moravia where the climatic and soil conditions are more favourable for the expansion of vineyards.
• The probability of further global warming and its changes expressed by climate indices are important for Czechia in the choice of cultivated vine varieties. Varieties intended for the production of red wines are already doing better in Czechia than in the past. That needs to be taken into account (Fig. 1).

#### 4.5.2 Implications for theory

• Monitor the development of the time series of the economic performance indicators of viticulture and grape vine growing in Czechia and, based on its statistical evaluation, recommend another suitable procedure for the sustainable development of the sector in Czechia.

### 4.6 Limitations of the research

The growing global concern with sustainability has driven companies to rethink their business model and seek new ways to operate and face this challenge [68]. This research study is based primarily on knowledge about the sustainable development of indicators of the economic performance of viticulture and grape vine growing in Czechia and their evaluation for the purposes of expanding of the area of vineyards in favour of varieties suitable for the production of red wines in Czechia.

It would undoubtedly be useful to find out the findings on the development of identical indicators in countries with similar soil conditions for grape vine growing, such as Germany or Austria. Then make a comparison with the research of wine experts in countries with similar vineyard locations, such as northern France, Germany and Austria, and verify it statistically. Above all, here in Europe, the application of our research model of the impact of climate change on regional viticulture and viticulture is possible.

The method of examining the compliance of the impact of climate change on the structure of wine production with the development of consumer preferences in a certain region is generally applicable. However, the prerequisite is the creation of climate maps of the monitored area and research into the structure of consumer demand for the given product - wine. The novelty of the method used lies not only in the expression of the impact of climate change on vine growing, but also its impact on the change in the structure of wine consumption in the respective region.

The previous claim about the influence of climate change on the structure and composition of the production of cultivated vines is also confirmed by the already mentioned study by Ashenfelter and Storchmann [25] “that climate change is likely to produce winners and losers, with the winners being those closer to the North and South Poles“.

## 5 Conclusion

Wine growers from the Czech Republic are fully competitive with top wines from other parts of Europe or the world. It is worth emphasizing the qualitative peculiarities and advantages of natural acids in the wines from local regions. Moravian and Czech wines are of high quality, diverse in varieties, they reflect the character of our climate and soil conditions, and each wine-maker imprints their unmistakable craft on them. After all, the above-average quality of Czech wines is also confirmed by regular successes at international competitions, where they win over the wines of the renowned wine powers. As our research has shown, climate change and related warming will lead to an increase in the area planted with varieties suitable for red wine production in the Czech Republic. The contradiction between the current popularity of white wines and the growing production of red wines will then lead to the need to import white wines from regions located further north.

The pride of Moravian viticulture was rather white varieties, but that is changing. Especially red wines, in which southern Europe has dominated so far, could have an exceptional quality here. This is thanks to, among other things, climate change. Over the last 60 years, the average temperature in Moravia has increased by one and a half degrees Celsius. Warming manifests itself mainly in autumn, when the grapes ripen. The alternation of warm days and cold nights is typical, which has an impact on higher amounts of aromatic substances.

Previous research shows that climate changes lead to a rise in air temperature, which significantly increases the water needs of plants. Climate change continues to pose a threat to the sustainability of water resources [69]. The vine harvest is often complicated by torrential rain, the yield per hectare, the spring frosts or the alternation of longer periods of drought or excessive rain, which then cause the formation of mould. Given factors have an influence on grape yields and their fluctuations; however, the warming of the atmosphere and the higher temperatures in the summer months have a positive effect on the quality of red wines, which have not done very well in the region in the past. Then again, a portion of the cultivating frameworks as of now situated in sweltering and dry territories are required to be most seriously influenced by environmental and climate change [70].

While climate change is a long-term challenge for Czech wine-makers, they have had to deal quickly with a pandemic and a lockdown, like many other companies in Europe. They bet on tastings and sales over the Internet and on the young generation of emerging wine growers. And many of them confirm that there was more interest in wine in the pandemic. On-line marketing is often the work of the sons and daughters of wine-makers, representatives of the emerging generation, among whom wine growers are seeing an increasing interest in quality wine.

## Author contribution statement

Aleksandre Petriashvili: Conceived and designed the experiments; Contributed reagents, materials; Wrote the paper. Jiří Mach: Performed the experiments; Contributed reagents, materials; Wrote the paper. Michal Štěbeták: Analyzed and interpreted the data; Wrote the paper. Marie Prášilová: Analyzed and interpreted the data; Wrote the paper. Roman Svoboda: Contributed reagents, materials, Wrote the paper. Miroslava Navrátilová: Performed the experiments; Wrote the paper. Markéta Beranová: Performed the experiments; Wrote the paper. Kamila Veselá: Analyzed and interpreted the data; Contributed reagents, materials. Václav Hofman: Analyzed and interpreted the data. Otakar Němec: Analyzed and interpreted the data.

## Data availability statement

Data included in article/supplementary material/referenced in article.

## Declaration of competing interest

The authors declare that they have no known competing financial interests or personal relationships that could have appeared to influence the work reported in this paper.
