# Supplementary material for: The impact of climate change on the sustainability of wine production and the structure of its consumption in Czechia
Source: Heliyon. 2023 Jul 3;9(7):e17882. doi: 10.1016/j.heliyon.2023.e17882 (PMC10395292; doi:10.1016/j.heliyon.2023.e17882)
Supplement: Multimedia component 1 [file mmc1.docx]

**I. Introductory Questions**

1) I am: a) male

b) female

2) Age category a) 0-20

b) 21-30

c) 31-40

d) 41-50

e) 51-60

f) 61 and over

3) Highest educational attainment a) Primary school

b) High school without high school diploma

c) High school with high school diploma

d) Higher vocational school

e) University

4) I live in the following region:

a) Capital City of Prague h) Karlovy Vary

b) Central Bohemian i) Hradec Králové

c) Pardubice j) South Bohemian

d) Zlín k) Olomouc

e) Liberec l) Vysočina

f) Moravian-Silesian m) Ústí nad Labem

g) Pilsen n) South-Moravian

5) Size of the municipality I live in:

a) 0-1,000 inhabitants d) 20,001 - 100,000 inhabitants

b) 1,001 - 5,000 inhabitants e) more than 100,000

c) 5,001 - 20,000 inhabitants

6) Average monthly net income

a) 0 - 15,000 d) 30,001-40,000

b) 15,001-20,000 e) 40,001-50,000

c) 20,001-30,000 f) more than 50,000

**II. Questions Focused on Wine Consumption**

1. Do you drink wine (even occasionally)?

(If not, proceed directly to question III.)

a) yes

b) no, I’m abstinent

2. How often do you indulge in wine? 1 glass of wine also counts.

a) daily

b) every other day

c) once a week

d) several times a week

e) several times a month

f) only occasionally

3. What colour of wine do you prefer?

a) white

b) rosé

c) red

4. Which wines do you prefer in terms of residual sugar content?

a) dry

b) semi-dry

c) semi-sweet

d) sweet

5. Which wines do you prefer in terms of their sugar content when the grapes are picked?

a) table wine

b) quality wine

c) wine with an attribute

d) the sugar content of the grapes is not important to me

6. Do you think that climate changes, which are also taking place in the Czech Republic, have an effect on the sugar content of grapes?

a) definitely yes

b) rather yes

c) I don’t know

d) rather not

e) definitely not

7. Which wines do you prefer in terms of country of origin?

a) foreign wines

which particular country do you prefer? Please write here:

b) Czech wines

do you have a favourite wine region? Please write here:

8. How important is the country of origin, wine region or specific winery to you when choosing a wine?

a) I only buy wines from proven wineries, preferred wine regions or a preferred country

b) I mainly buy wines from proven wineries, preferred wine regions or a preferred country

c) I often buy wines from proven wineries, preferred wine regions or a preferred country, but I also try other wines

d) country of origin, wine region or specific winery does not play a role in my choice of wine

9. Do you have any idea how much sugar a glass of dry wine contains on average?

a) 1-2 grams of sugar

b) 3-6 grams of sugar

c) 10-15 grams of sugar

d) more than 15 grams of sugar

10. Do you have any idea how many calories a glass of dry wine contains?

a) 50 kcal

b) 100 kcal

c) 150 kcal

d) more than 200 kcal

11. Where do you most often buy wine?

a) directly from the wine maker

b) wine shop

c) supermarket

12. have you bought or do you buy fair-trade wines?

a) I don’t know what fair-trade is

b) yes, once

c) yes, sometimes I buy these wines

d) yes, I buy these wines regularly

e) never

13. In what price range do you most often buy wine?

a) up to 70 CZK

b) 71-100 CZK

c) 101-200 CZK

d) 201-300 CZK

e) more than 301 CZK

14. When buying wine, you prioritize:

a) price (e.g. also a discount event)

b) quality (sugar content of grapes at harvest)

c) specific winery, wine region or country

d) appearance of the bottle and label

**III. Questions Focused on Behaviour in an Emergency Situation.**

Please indicate the level of your agreement with the following statements regarding shopping behaviour in an emergency situation (restrictions related to COVID 19).

1. I buy larger quantities of food just to be safe. *

I completely disagree

I disagree

I can’t decide

I agree

I completely agree

2. I buy more bottles of wine just to be safe. *

I completely disagree

I disagree

I can’t decide

I agree

I completely agree

3. I don’t think my shopping behaviour has changed, I don’t stock up on food more than usual. *

I completely disagree

I disagree

I can’t decide

I agree

I completely agree

4. I don’t think my shopping behaviour has changed, I don’t stock up on wine more than usual. *

I completely disagree

I disagree

I can’t decide

I agree

I completely agree

5. I try not to panic and not to buy food I don’t need. *

I completely disagree

I disagree

I can’t decide

I agree

I completely agree

6. I also buy non-perishable foods that I don’t normally buy. *

I completely disagree

I disagree

I can’t decide

I agree

I completely agree

7. I don’t stock up on any food. *

I completely disagree

I disagree

I can’t decide

I agree

I completely agree

8. I don’t stock up on any wine. *

I completely disagree

I disagree

I can’t decide

I agree

I completely agree

9. I bought food for stock that will last at least 2 weeks. *

I completely disagree

I disagree

I can’t decide

I agree

I completely agree

10. I go grocery shopping less often than in a normal situation. *

I completely disagree

I disagree

I can’t decide

I agree

I completely agree

11. I go shopping for alcohol (wine) less often than in a normal situation. *

I completely disagree

I disagree

I can’t decide

I agree

I completely agree

12. It upsets me when I don’t get food that is commonly available under normal circumstances. *

I completely disagree

I disagree

I can’t decide

I agree

I completely agree

13. If I see that there won’t be certain food left for other people, I prefer to take fewer pieces. *

I completely disagree

I disagree

I can’t decide

I agree

I completely agree

14. I prefer to take more pieces of selected food items as they can sell out quickly. *

I completely disagree

I disagree

I can’t decide

I agree

I completely agree

15. I don’t care if other people have built up stocks. *

I completely disagree

I disagree

I can’t decide

I agree

I completely agree

16. It is important to me that I have built up stocks for myself and my family, after all everyone has to figure it out for themselves. *

I completely disagree

I disagree

I can’t decide

I agree

I completely agree

17. It is important to me that I have built up stocks of wine for myself and my family, after all everyone has to figure it out for themselves. *

I completely disagree

I disagree

I can’t decide

I agree

I completely agree

18. I always have non-perishable food in stock at home. *

I completely disagree

I disagree

I can’t decide

I agree

I completely agree

19. I always have bottles of wine in stock at home. *

I completely disagree

I disagree

I can’t decide

I agree

I completely agree

20. I am not afraid that there will be a shortage of food in the market. *

I completely disagree

I disagree

I can’t decide

I agree

I completely agree

21. I am not afraid that there will be a shortage of wine in the market. *

I completely disagree

I disagree

I can’t decide

I agree

I completely agree

22. I believe that drinking wine has helped me in a difficult life situation during the Covid19 pandemic.

I completely disagree

I disagree

I can’t decide

I agree

I completely agree
